# Long-term renal outcome in patients with malignant hypertension: a retrospective cohort study

**DOI:** 10.1186/1471-2369-13-71

**Published:** 2012-07-30

**Authors:** Fouad Amraoui, Sarah Bos, Liffert Vogt, Bert-Jan van den Born

**Affiliations:** 1 Departments of internal and vascular medicine, Academic Medical Centre, Amsterdam, The Netherlands; 2 Departments of internal medicine and nephrology, Academic Medical Centre, Meibergdreef 9, Amsterdam 1105 AZ, The Netherlands

**Keywords:** Malignant hypertension, End-stage renal disease, Chronic kidney disease, Renal outcome, Mortality

## Abstract

**Background:**

Malignant hypertension is frequently complicated by renal insufficiency. Although the survival of this hypertensive emergency has improved, recent data on renal outcome and its predictors are lacking. We assessed renal outcome and its predictors in patients with malignant hypertension.

**Methods:**

Retrospective analysis of patients admitted with malignant hypertension in Amsterdam, the Netherlands between August 1992–January 2010. Follow-up data on vital status, renal function and blood pressure (BP) were obtained from the outpatient department and from general practitioners. The primary composite endpoint was end-stage renal disease (ESRD) defined as the start of kidney replacement therapy (KRT) or ≥ 50% decline of estimated glomerular filtration rate (eGFR). The secondary endpoint was all cause mortality.

**Results:**

A total of 120 patients admitted with malignant hypertension were included. After a median follow-up period of 67 months (IQR 28 to 108 months) the primary endpoint was reached by 37 (31%) patients, whereas 18 patients (15%) reached the secondary endpoint. Twenty-nine (24%) patients started KRT and 8 patients (7%) had an eGFR decline ≥ 50%. After the acute phase (> 3 months after admission), initial serum creatinine and follow-up BP were the main predictors of future ESRD with hazard ratios of 6.1 (95% CI, 2.2–17) for patients with initial serum creatinine ≥ 175 μmol /L and 4.3 (95% CI, 1.4–14) for patients with uncontrolled hypertension.

**Conclusions:**

Progressive renal function decline leading to ESRD remains a major threat to patients with malignant hypertension. BP control during follow-up was an important modifiable predictor of renal outcome.

## Background

Malignant hypertension is a hypertensive emergency usually defined by the presence of severe hypertension in combination with ischemic retinal changes consistent with grade III or IV hypertensive retinopathy according to the Keith, Wagener and Barker classification [1]. Malignant hypertension is frequently complicated by renal dysfunction [2,3]. In most cases this is secondary to malignant hypertension with renal biopsy specimens typically showing acute ischemic changes secondary to medial hypertrophy, intimal hyperplasia and fibrinoid necrosis of small arteries and arterioles [4]. Although the survival of malignant hypertension has considerably improved with the advent of antihypertensive therapy, end-stage renal disease (ESRD) remains a significant cause of morbidity and mortality [5,6]. Depending on the setting and patient characteristics between 18% and 41% of patients may require kidney replacement therapy (KRT) during the acute phase [6,7].

One would expect that because of advances in the awareness, treatment and control of hypertension in the population at large the incidence of malignant hypertension and its renal complications would have declined. However, we and others have shown that malignant hypertension remains relatively common in large multi-ethnic communities and in urban sub-Saharan African populations. In the Netherlands, the relative contribution of malignant hypertension to the total number of patients starting KRT has increased by 40% in the past two decades [8]. Annual reports of the European Dialysis and Transplant Association and the US Renal Data System show that hypertension is an important cause of ESRD, but the role of hypertensive crises are not specified [9,10].

Previous studies have demonstrated recovery from acute renal dysfunction in some patients with malignant hypertension and ESRD [11,12]. However, data concerning long-term renal outcome are limited. Moreover, the influences of modifiable predictors such as blood pressure (BP) control have not been established. In this study, we aimed to assess long-term renal outcome and predictors of ESRD and renal function decline in patients with malignant hypertension.

## Methods

Included were patients presenting with malignant hypertension at a large teaching hospital, serving a multi-ethnic community in Amsterdam, the Netherlands. Patients were recruited between August 1992 and January 2010. The methods for the selection strategy have been previously described [6]. Briefly, the hospital database, in which the diagnosis at discharge is recorded according to the International Classification of Diseases codes (ICD) was searched. All charts of patients admitted with the diagnosis ‘essential malignant hypertension’ (ICD 401.0), ‘secondary malignant hypertension’ (ICD 405.09), ‘hypertension with cardiac disease/ malignant’ (ICD 402.0), ‘hypertension with kidney disease/malignant’ (ICD 403.0) and ‘hypertensive encephalopathy’ (ICD 437.2), were reviewed for the WHO criteria of malignant hypertension (i.e. diastolic BP ≥ 120 mmHg and presence of grade III or IV hypertensive retinopathy [1]. To identify the presence of registration errors, computer data of all patients discharged with the diagnosis ‘essential hypertension’ (ICD 401.9) were also analyzed which showed the presence of one patient with malignant hypertension. Hereafter, we subjected our selection strategy to a sensitivity analysis by searching the emergency room archives for patients fulfilling the clinical criteria for malignant hypertension in a sample of 3 different years, with each year randomly selected from a period of 5 consecutive years between 1992-2008.

Excluded were patients < 18 years, pregnant women and patients who were already on dialysis before admission and patients referred from elsewhere. This study was performed in adherence with the Declaration of Helsinki. The Ethics Committee in our hospital decided approval was not required.

### Definitions and analyses

Follow-up and outcome data were obtained from the outpatient department and from general practitioners. The primary endpoint was ESRD defined as a composite of the start of permanent KRT or a 50% reduction in eGFR during follow-up. All cause mortality was assessed as secondary endpoint. Because both recovery and deterioration of renal function occur frequently during the initial treatment of malignant hypertension [6], renal outcome data were censored during the first 3 months following admission. Change in eGFR was calculated as the difference between first serum creatinine 3 months after admission and last available serum creatinine during follow-up. For calculating eGFR, the Modification of Diet in Renal Disease (MDRD) formula was used [13]. Vital status of patients who were lost to follow-up was assessed by inquiry of the municipal administration registries.

Ethnicity was defined as self-reported black or self-reported white. Black participants were mainly from sub-Saharan Africa and from Surinam or the Dutch Antilles. Secondary causes of malignant hypertension were reported as present in case of any medical condition or therapy that could be related to the development of this type of hypertensive emergency. Thrombotic microangiopathy (TMA) was defined as 1) thrombocytopenia (platelet count < 150 x10E9/L) together with either an elevated lactate dehydrogenase (LDH ≥ 220 U/L) or the presence of fragmentocytes and 2) resolution of these parameters with BP lowering therapy. Macroalbuminuria was defined as urinary protein excretion greater than 300 mg/24-h, > 200 mg/L in spot urine or 2+ for dipstick proteinuria. Left ventricular hypertrophy was considered present when detected by a cardiologist on cardiac ultrasound (left ventricular wall thickness > 11 mm) or by ECG as defined by the Sokolow-Lyon criteria. Mean follow-up BP was defined as the average of three BP recordings starting with the first measurement 3 months after admission. The second BP recording was the measurement closest to the median of the follow-up period and the third recording was defined as the last available follow-up BP value. Blood pressure was considered to be adequately controlled if the average of all measurements was < 140/90 mmHg.

### Statistical analysis

Data are expressed as mean +/- standard deviation (SD) when normally distributed and as median and interquartile range (IQR) when distribution was skewed. Frequencies and percentages are given for categorical variables. Between group differences were assessed by *t*-test for parametric and Mann–Whitney *U* test for non-parametric distributions. Chi-square analysis was used for categorical variables. Cox proportional hazard analysis was used to assess renal outcome predictors during follow-up. First, single variables with a pathophysiological relevance that had a significant association with the outcome parameter were entered in the model. Next, a backward elimination method was used in which the variable with the smallest partial correlation with the dependent variable was removed first. Hazard ratios (HR) and 95% confidence intervals (CI) were calculated. Finally, significant variables were tested for interaction. For statistical analyses, SPSS software was used (Statistical Package for the Social Sciences, version 18.0, Inc. Chicago, Illinois, USA). P values were considered to indicate a significant difference if *P* < 0.05.

## Results

### Baseline characteristics

**Figure 1:**
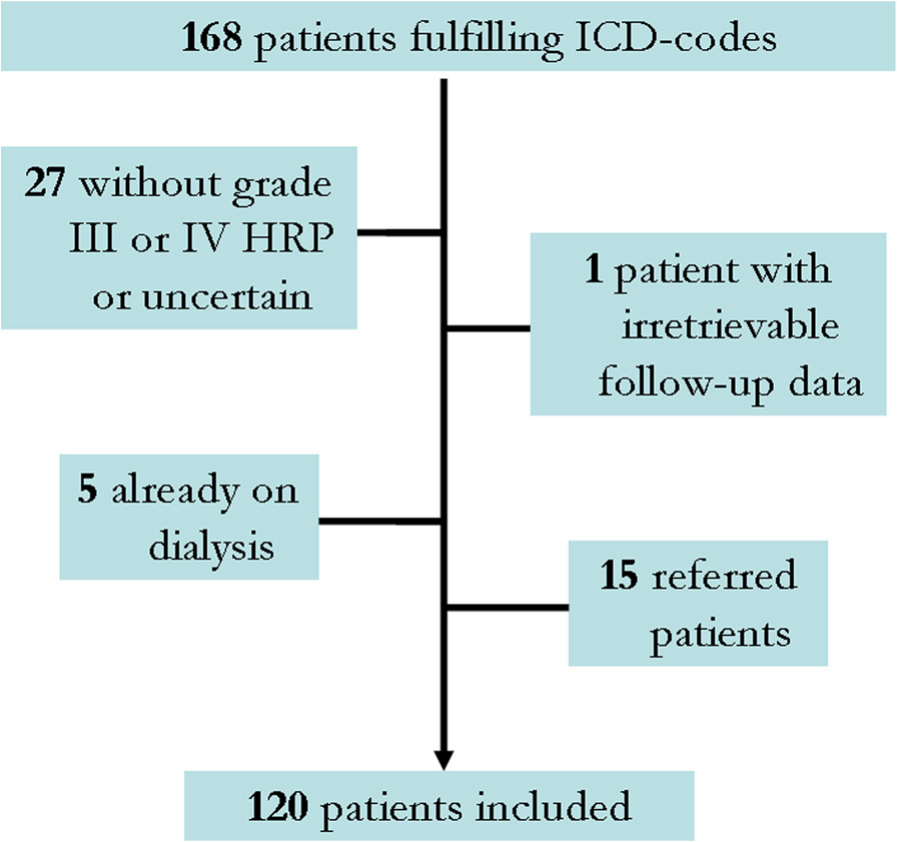
**Patient selection.** HRP indicates hypertensive retinopathy.

A total of 168 patients were identified via the hospital database search. Of these patients, 120 fulfilled the WHO criteria of malignant hypertension and could be included in the study (Figure 1). A sensitivity analysis showed that no patients who visited the emergency room with malignant hypertension in 3 randomly selected years between 1992-2008 were missed.

Baseline characteristics are summarized in Table 1. Median serum creatinine at admission was 175 μmol /L (IQR 104-402 μmol /L). Patients with serum creatinine ≥ 175 μmol /L were more often black (59% vs. 36%, *P* = 0.01) and had higher systolic (235 vs. 225 mmHg, *P* = 0.03) and diastolic BP values (148 vs. 141 mmHg, *P* = 0.04). These patients more frequently displayed TMA (51% vs. 9%, *P* < 0.001), macroalbuminuria (92% vs. 53%, *P* < 0.001) and left ventricular hypertrophy (89% vs. 70%, *P* = 0.01). There was no difference in age, sex, severity of retinopathy or the presence of primary renal and renovascular disease. At the start of follow-up (3 months after admission) median eGFR was 41 ml/min/1.73 m^2^ (IQR 20-68 ml/min/1.73 m^2^).

**Table 1:** Baseline characteristics of 120 consecutive patients with malignant hypertension

**Baseline characteristics**	
Male, n (%)	83 (69)
Age, mean (SD)	44 (12)
Black, n (%)	57 (48)
Systolic blood pressure (mmHg), mean (SD)	230 (23)
Diastolic blood pressure (mmHg), mean (SD)	145 (17)
Previous hypertension, n (%)	65 (54)
Use of anti-hypertensive medication, n (%)	39 (33)
Secondary cause of malignant hypertension, n (%)	25 (21)
Serum creatinine at admission (μmol /L), median (IQR)	175 (104–402)
Thrombotic microangiopathy, n (%)	36 (30)
Hypertensive encephalopathy	11 (9)
Retinopathy grade IV, n (%)	66 (55)
Left ventricular hypertrophy, n (%)	95 (79)
Macroalbuminuria, n (%)	66 (55)
Diabetes mellitus type 2, n (%)	6 (5)
Current smoker, n (%)	39 (33)

*IQR indicates interquartile range.

Primary renal disease could be identified in 9 patients (8%), whereas renovascular disease could be identified in 7 patients (6%). Primary renal diagnoses included biopsy proven membranoproliferative glomerulonephritis (3 patients), IgA nephropathy (3 patients) and renal involvement of systemic scleroderma (1 patient). Polycystic kidney disease (1 patient) and reflux nephropathy (1 patient) were diagnosed radiologically. Renovascular diseases included renal artery stenosis (4 patients), renal artery obstruction (1 patient), renal infarction (1 patient) and renovascular abnormalities secondary to polyarteritis nodosa (1 patient).

### Renal outcome and mortality

After a median follow-up of 67 months (IQR 28–108 months), 31% reached the primary composite endpoint, 29 patients (24%) started KRT, whereas 8 patients (7%) had an eGFR decline ≥50% compared to baseline. Sixteen patients (13%) required haemodialysis within 3 months after admission and this could be discontinued in 2 patients within this period. Of the 14 patients who reached the primary endpoint within 3 months after admission, 10 patients were black and 4 were white (*P* = 0.06). The proportion of patients developing ESRD was not significantly different between those having primary malignant hypertension and those with primary renal or renovascular disease (24% vs. 38% respectively, *P* = 0.18). A total of 18 (15%) patients died during follow-up, none of the patients died within 3 months after admission. Death was caused by cardiovascular events (6 patients), malignancy (3 patients), ESRD (2 patients) and infectious diseases (2 patients). The cause of death of 5 patients was uncertain.

### Predictors of long-term renal outcome and mortality

For analysis of predictors of long-term renal outcome and mortality, patients requiring permanent haemodialysis within 3 months after admission were censored, leaving 106 patients for analysis. Follow-up BP was adequately controlled in 33 out of 92 patients (36%) and uncontrolled in 59 (64%) patients. Black patients had a higher follow-up BP compared to white patients with a systolic BP of 153 ± 23 mmHg in black and 140 ± 21 mmHg in white patients (*P* < 0.01). Diastolic BP was 96 ± 12 mmHg versus 85 ± 11 mmHg respectively (*P* < 0.01). Data on BP during follow-up were incomplete for 14 out of 106 (13%) patients. Antihypertensive treatment during follow-up consisted of a mean number of 3.3 ± 1.1 drugs. Patients with controlled follow-up BP used on average 2.9 ± 1.1 antihypertensive drugs, while patients with uncontrolled BP used on average 3.5 ± 1.1 drugs (*P* < 0.01).

**Figure 2:**
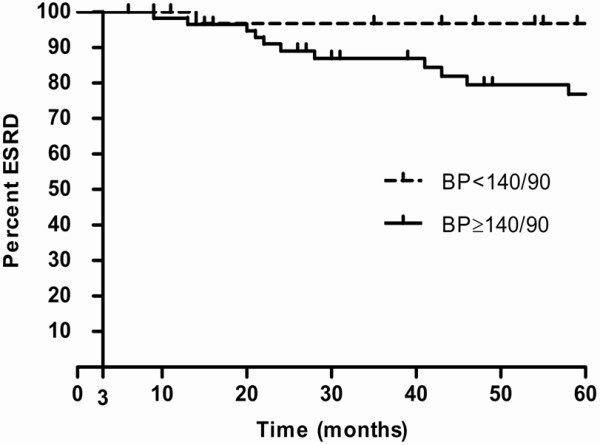
**ESRD in patients with malignant hypertension having blood pressure <140 / 90 mmHg and ≥140 / 90 mmHg during follow-up.** Kaplan-Meier survival plot of ESRD during follow-up. ESRD was defined as a composite of the start of kidney replacement therapy or a 50% reduction in eGFR.

The primary endpoint was reached by 23 out of 106 (22%) patients after a median follow-up of 67 months (IQR 28-108 months) with 15 (14%) patients starting KRT and 8 (8%) patients with an eGFR decline ≥ 50% (Table 2). Initial serum creatinine ≥ 175 μmol /L and uncontrolled hypertension (BP ≥ 140/90 mmHg) during follow-up were identified as main predictors of the composite renal outcome (Figure 2) with hazard ratios (HR) of 6.1 (95% CI, 2.2–17) and 4.3 (95% CI, 1.4–14) respectively (Table 3). Patients with a mean follow-up BP > 160/100 mmHg had a HR of 5.1 (95% CI, 1.4–18) for developing ESRD (*P* = 0.01).

**Table 2:** Patient characteristics at follow-up

**Follow-up characteristics**	
Follow-up time, months, median (IQR )*	67 (28-108)
Died, n (%)	18 (15)
Start of kidney replacement therapy, n (%)	29 (24)
50 % decline of eGFR, n (%)	8 (7)
Systolic blood pressure (mmHg), mean (SD)	146 (23)
Diastolic blood pressure (mmHg), mean (SD)	90 (13)
Blood pressure < 140/ 90 mmHg, n (%) †	33 (36)
BP 140-160/90-100 mmHg, n (%)	42 (46)
BP 160-180/100-110 mmHg, n (%)	12 (13)
BP > 180/100 mmHg, n (%)	5 (5)
ACE-inhibitor or ARB, n (%)	74 (80)
Beta-blocking agent, n (%)	61 (66)
Calcium-antagonist, n (%)	79 (86)
Diuretic, n (%)	64 (70)
Alfa-blocking agent, n (%)	18 (20)

*IQR indicates interquartile range.

† Data on hypertension status and antihypertensive medication during follow-up were available for 92 patients. Percentages are calculated for a total of 92 patients. One patient used Minoxidil in addition to all 5 antihypertensive classes listed in the table.

**Table 3:** Cox regression analyses using backward elimination method to predict indicators for ESRD during follow-up

**Variables**	**Rank**†	**P-value**	**HR**	**95% CI**
Age	5	0.60	0.9	0.5-1.6
Male	3	0.10	2.5	0.8-7.7
Black	6	0.82	1.1	0.3-3.8
Initial creatinine **≥** 175 μmol /L	1	0.00	6.1	2.2-17
Uncontrolled hypertension	2	0.01	4.3	1.4-14
Trombotic microangiopathy	4	0.19	1.9	0.7-5.2

* Renal outcome was defined as a composite of the start of KRT or 50% decline of eGFR.

†Indicates the rank of elimination in stepwise backward Cox regression.

Adjustment for primary renovascular and kidney disease did not materially change the predictive value of hypertension control on renal outcome. After adjustment HR were 5.2 (95% CI 1.8-15) for patients with initial serum creatinine ≥ 175 μmol /L and 4.7 (95% CI, 1.3–17) for patients having an average follow-up BP ≥ 140/90. The interaction between serum creatinine and hypertension status was not significant (*P* = 0.45). Initial serum creatinine ≥ 175 μmol /L and uncontrolled hypertension during follow-up were also the main predictors of mortality with HR of 6.3 (95% CI, 2.0–20) and 3.1 (95% CI, 1.0–9.9) respectively.

## Discussion

In this study, we show that ESRD remains highly prevalent among patients who were previously admitted with malignant hypertension, with 31% starting KRT or having a 50% eGFR decline after a median follow-up of 67 months. Major determinants of long-term renal outcome were initial serum creatinine values and BP control during follow-up. Only one controlled hypertensive patient developed the renal endpoint during follow-up as opposed to 22 patients with uncontrolled hypertension. Although previously identified as an important predictor of renal function improvement [6], the presence of TMA was not found to predict ESRD.

The incidence rates of ESRD in series describing patients with malignant hypertension vary from 11% to 53% [2,14,15]. These variations may be due to differences in follow-up time, population characteristics and selection bias. To prevent selection bias, we analyzed only patients admitted via the emergency room and excluded referred patients with malignant hypertension as they could be more likely to have ESRD. In addition, because our aim was to assess predictors of long-term renal outcome, we censored data of the first 3 months after admission as renal function during this period is frequently not yet stabilized.

This study demonstrates that the risk of developing ESRD in patients with malignant hypertension is markedly elevated. For example in the MRFIT trial, only 3% of patients with severe non-malignant hypertension (BP ≥ 210/120 mmHg) developed ESRD after 16 years [16]. However, patients with evidence of end-organ damage including renal dysfunction (serum creatinine > 177 μmol/L) were excluded from the MRFIT trial. Therefore, differences in baseline renal function may account for the much higher incidence of ESRD in patients with malignant hypertension, who by definition display evidence of end-organ damage including renal damage. Besides causing acute renal damage, malignant hypertension may also reflect difficulty to regulate blood pressure in individuals on the long-term as placebo-controlled randomized clinical trials performed decades ago, showed that antihypertensive treatment effectively prevented progression of essential hypertension to malignant hypertension [17,18]. In addition, non-compliance to antihypertensive treatment and the lack of insurance and access to primary health care was observed more frequently in patients with severe and malignant hypertension [19]. In a previous report we showed that non-compliance to antihypertensive treatment and lack of medical insurance were also observed frequently in our population, particularly among ethnic minorities [3]. Our observation that patients with uncontrolled BP on follow-up had more antihypertensive drugs compared to those with controlled hypertension could be an indication of non-compliance. However, average BP was calculated for the complete follow-up period, whereas data on antihypertensive drugs represent the last known treatment. The higher number of antihypertensive drugs could therefore also reflect an effort to control higher BP values earlier in the follow-up period.

Finally, in addition to differences in baseline renal function and long-term blood pressure control, the increased risk of developing ESRD in malignant hypertension might be attributable to endothelial dysfunction. While having similar blood pressure values, patients who previously suffered from malignant hypertension have been shown to display more pronounced endothelial dysfunction as compared to patients with high-risk (non-malignant) hypertension [20].

The potential effect of BP control on long-term renal outcome in malignant hypertension has been described previously in two small case-series from the 1980s including a total of 14 patients [21,22]. However, as BP control was achieved in almost all patients in these two case-series the potential benefit of BP lowering therapy on progression of ESRD could not be established. In our series, patients with uncontrolled hypertension (BP ≥ 140/90 mmHg) had a 4.3 fold higher risk of developing ESRD compared to patients in whom BP was controlled. Patients with more severe hypertension (> 160/100 mmHg) during follow-up had a 5.1 fold higher risk of ESRD. It is conceivable that the propensity to develop ESRD in those with uncontrolled hypertension may be due to hyperperfusion of remaining nephrons in those with renal damage during the acute phase. This is also exemplified by the fact that renal function, while often improving after the initial phase, does not fully recover [6], suggesting residual renal damage.

There was no interaction between hypertension status and renal function at admission suggesting that the presence of renal dysfunction was not an important determinant of hypertension status. Several studies including our own have outlined the potential recovery of renal function in patients who were admitted with malignant hypertension after institution of BP lowering therapy [11,12,23]. In the current analysis, the presence of TMA as evidence of vascular damage, did not predict long-term renal outcome suggesting that the detrimental, but potentially reversible effect of TMA had no negative effect on long-term renal outcome.

This study has both strengths and limitations. Strengths include the contribution of clinically relevant and previously unavailable data on long-term renal outcome of an unselected, well described and relatively large cohort of patients with malignant hypertension. Limitations include its retrospective nature and consequently the possibility of coding errors. However, a sensitivity analysis showed that no patients with malignant hypertension who visited the emergency room between 1992–2008 were missed. Secondly, follow-up data on BP control were incomplete for 14 out of 106 patients (13%), which may have influenced the association between hypertension status and ESRD. However baseline characteristics of patients who lacked complete BP data were not different with regard to initial serum creatinine, blood pressure values and need for KRT (data not shown). Thirdly, analysis of the predictive value of follow-up proteinuria in this study was hampered by differences in testing methods for urinary protein excretion. Also, the reason for performing urine analysis in the follow-up was subject to bias by indication, as it was more frequently done in patients showing progressive renal function decline. Finally, a separate analysis could not be performed for patients with primary renovascular and kidney disease because of the limited number of patients. However, Cox regression analysis revealed similar results after exclusion of these patients (data not shown). Although ESRD did not occur significantly more often in patients with than without renovascular and kidney disease, this study may have been underpowered to demonstrate clinically relevant differences in renal outcome.

## Conclusions

Patients with malignant hypertension display a markedly elevated risk for developing ESRD after the acute phase. BP control during follow-up was strongly associated with the risk of progression to ESRD, prompting to adequate blood pressure regulation for improving long-term renal outcome in these patients.

## Competing interests

The authors declare that they have no competing interests.

## Authors' contributions

BJB was responsible for the conception and design of this study and revision of the manuscript. LV contributed to the analysis and interpretation of data and revision of the manuscript. SB and FA equally contributed to data collection, data analysis and drafting of the manuscript. All authors have read and approved the final manuscript.

## Pre-publication history

The pre-publication history for this paper can be accessed here:

http://www.biomedcentral.com/1471-2369/13/71/prepub

## References

[B1] Keith NM Wagener HP Barker NW Some different types of essential hypertension: their course and prognosis Am J Med Sci 1939 196 332 343 10.1097/00000441-197412000-00004 4616627

[B2] Lip GY Beevers M Beevers DG Complications and survival of 315 patients with malignant-phase hypertension J Hypertens 1995 13 915 924 10.1097/00004872-199508000-00013 8557970

[B3] van den Born BJ Koopmans RP Groeneveld JO van Montfrans GA Ethnic disparities in the incidence, presentation and complications of malignant hypertension J Hypertens 2006 24 2299 2304 10.1097/01.hjh.0000249710.21146.38 17053554

[B4] Sanerkin NG Vascular lesions of malignant essential hypertension J Pathol 1971 103 177 184 10.1002/path.1711030306 5567174

[B5] Lane DA Lip GY Beevers DG Improving survival of malignant hypertension patients over 40 years Am J Hypertens 2009 22 1199 1204 10.1038/ajh.2009.153 19696746

[B6] van den Born BJ Honnebier UP Koopmans RP van Montfrans GA Microangiopathic hemolysis and renal failure in malignant hypertension Hypertension 2005 45 246 251 10.1161/01.HYP.0000151620.17905.ee 15596574

[B7] Kadiri S Olutade BO The clinical presentation of malignant hypertension in Nigerians J Hum Hypertens 1991 5 339 343 1956031

[B8] Dutch Renal Registry (RENINE) 2010 https://www.renine.nl/page?id=home&lang=en

[B9] Collins AJ Foley RN Herzog C Chavers B Gilbertson D Ishani A Kasiske B Liu J Mau LW McBean M US Renal Data System 2010 Annual Data Report Am J Kidney Dis 2011 57 A8 e1-A8 526 10.1053/j.ajkd.2010.10.007 21184928

[B10] Stel VS van de Luijtgaarden MW Wanner C Jager KJ The 2008 ERA-EDTA Registry Annual Report-a precis NDT Plus 2011 4 1 13 10.1093/ndtplus/sfq191 21245934 PMC3022422

[B11] Mourad G Mimran A Mion CM Recovery of renal function in patients with accelerated malignant nephrosclerosis on maintenance dialysis with management of blood pressure by captopril Nephron 1985 41 166 169 10.1159/000183574 3900778

[B12] Isles CG McLay A Jones JM Recovery in malignant hypertension presenting as acute renal failure Q J Med 1984 53 439 452 6515000

[B13] Levey AS Bosch JP Lewis JB Greene T Rogers N Roth D A more accurate method to estimate glomerular filtration rate from serum creatinine: a new prediction equation. Modification of Diet in Renal Disease Study Group Ann Intern Med 1999 130 461 470 10075613 10.7326/0003-4819-130-6-199903160-00002

[B14] Guerin C Gonthier R Berthoux FC Long-term prognosis in malignant or accelerated hypertension Nephrol Dial Transplant 1988 3 33 37 3132637

[B15] Gonzalez R Morales E Segura J Ruilope LM Praga M Long-term renal survival in malignant hypertension Nephrol Dial Transplant 2010 25 3266 3272 10.1093/ndt/gfq143 20299339

[B16] Klag MJ Whelton PK Randall BL Neaton JD Brancati FL Ford CE Shulman NB Stamler J Blood pressure and end-stage renal disease in men N Engl J Med 1996 334 13 18 10.1056/NEJM199601043340103 7494564

[B17] Effects of treatment on morbidity in hypertension. Results in patients with diastolic blood pressures averaging 115 through 129 mmHg JAMA 1967 202 1028 1034 4862069

[B18] Effects of treatment on morbidity in hypertension. II. Results in patients with diastolic blood pressure averaging 90 through 114 mmHg JAMA 1970 213 1143 1152 4914579

[B19] Shea S Misra D Ehrlich MH Field L Francis CK Predisposing factors for severe, uncontrolled hypertension in an inner-city minority population N Engl J Med 1992 327 776 781 10.1056/NEJM199209103271107 1501654

[B20] Shantsila A Dwivedi G Shantsila E Butt M Beevers DG Lip GY Persistent macrovascular and microvascular dysfunction in patients with malignant hypertension Hypertension 2011 57 490 496 10.1161/HYPERTENSIONAHA.110.166314 21263115

[B21] Mitchell HC Graham RM Pettinger WA Renal function during long-term treatment of hypertension with minoxidil: comparison of benign and malignant hypertension Ann Intern Med 1980 93 676 681 7212474 10.7326/0003-4819-93-5-676

[B22] Nicholson GD Long-term survival after recovery from malignant nephrosclerosis Am J Hypertens 1988 1 73 75 3370137 10.1093/ajh/1.1.73

[B23] Cordingley FT Jones NF Wing AJ Hilton PJ Reversible renal failure in malignant hypertension Clin Nephrol 1980 14 98 103 7408261

